# Challenging stigma attached to mental disorders: A psychosocial perspective

**DOI:** 10.1192/j.eurpsy.2021.92

**Published:** 2021-08-13

**Authors:** A. Fiorillo

**Affiliations:** Department Of Psychiatry, University of Campania “L. Vanvitelli”, Naples, Italy

**Keywords:** discrimination, social inclusion, Stigma, Mental disorders

## Abstract

Stigma attached to mental disorders represent one of the main obstacles to patients’ full recovery and empowerment. In the last decades, many anti-stigma programmes have been implemented worldwide, but stigma still represents a major obstacle for people with severe mental disorders, their family members, friends and also healthcare professionals. Stigma is a complex social phenomenon, which entails a lack of knowledge, discriminating attitudes and excluding behaviours in the general population, which deserves a multi-level approach. In particular, anti-stigma strategies combining the three most common approaches, including contact, education, and organization of protest activity, are the most effective. Interventions should contain age-appropriate information and should be provided at an early age (e.g., in schools). Interdisciplinary approaches are recommended. In particular, contact strategies are important to reduce prejudice and change attitudes towards people with mental disorders and may be implemented either by video (interviews/personal testimonies), but ideally in person with affected individuals, reporting their real life experiences. In this workshop, the role of advocacy associations together with all stakeholders of mental health will be discussed in the process of fighting stigma according to a psychosocial perspective.

**Disclosure:**

No significant relationships.

